# Real-Time Kinematic Reconstruction of Human Lower Limbs Using a 3-IMU Wearable Sensor Network, Transformer Model, and Deployable Edge Computing

**DOI:** 10.3390/s26123706

**Published:** 2026-06-10

**Authors:** Yang Yu, Wei Dong, Hui Dong, Wenda Wang, Yongzhuo Gao, Dongmei Wu, Weiqi Lin

**Affiliations:** State Key Laboratory of Robotics and System, Harbin Institute of Technology, Harbin 150001, China; 24s008036@stu.hit.edu.cn (Y.Y.); dongwei@hit.edu.cn (W.D.); dongh@hit.edu.cn (H.D.); wangwenda@stu.hit.edu.cn (W.W.); gaoyongzhuo@hit.edu.cn (Y.G.); wdm@hit.edu.cn (D.W.)

**Keywords:** wearable sensors, inertial measurement unit (IMU), kinematic reconstruction, edge computing, human motion capture

## Abstract

Continuous monitoring of lower-limb kinematics in natural environments is essential for gait analysis and rehabilitation but remains challenging due to the limitations of optical systems and the inaccuracy of sparse inertial sensor methods. To address this, we propose a high-precision, minimalist wearable system utilizing only three inertial measurement units placed on the pelvis and shanks. In the data preprocessing stage, engineering modifications are made based on the traditional gradient descent algorithm to implement adaptive channel adjustment on the acceleration and magnetic data of a single IMU, aiming to alleviate the impact of motion acceleration and external magnetic interference on the temporal feature manifold. Subsequently, a pure Transformer neural network is utilized to capture long-range temporal dependencies, reconstructing full lower-limb kinematics without relying on rigid biomechanical assumptions. The model was optimized and deployed on an STM32N647 microcontroller to achieve real-time edge inference with a low latency of approximately 17 ms. Experimental results demonstrate that the proposed method achieves a mean absolute error of 2.41° for level walking, significantly outperforming traditional constrained Kalman filter approaches. Furthermore, it maintains high tracking robustness during complex nonlinear movements such as squatting and lunging. In conclusion, this edge-computing-enabled framework provides an accurate, comfortable, and real-time solution for unconstrained human motion capture in daily scenarios.

## 1. Introduction

Lower-limb kinematic reconstruction is a fundamental approach for the clinical diagnosis of movement disorders, gait analysis, and the evaluation of surgical outcomes and rehabilitation progress [1,2]. Currently, optical motion capture (OMC) systems, represented by Nokov [3] and Vicon [4], are widely regarded as the “gold standard” in the field due to their millimeter-level spatial accuracy. However, these systems are not only expensive but also constrained by camera fields of view, restricting experiments to highly controlled laboratory environments.

To bridge this gap, commercial wearable inertial systems, such as Xsens, have been developed and widely applied in human motion analysis [5]. However, these systems typically require a dense array of sensors (e.g., full-body suits with 17 IMUs), which is cumbersome for unconstrained daily wear. Therefore, developing a portable lower-limb motion capture system that can operate beyond laboratory constraints and enable all-day, multi-scenario monitoring has become an urgent need in biomechanics and rehabilitation medicine [6,7]. Nevertheless, achieving high-precision kinematic reconstruction using sparse inertial measurement unit (IMU) signals in complex real-world environments remains a significant challenge. To enable unconstrained gait monitoring, wearable inertial sensing technologies have made significant progress in recent years; however, substantial gaps remain between theoretical models and practical applications.

Early research on reconstruction from sparse sensors (RSC) predominantly relied on model-based approaches grounded in biomechanical constraints. Salarian et al. modeled the leg as a double pendulum and derived joint angles via linear regression [8]; Hu et al. employed a 3R gait model combined with inverse kinematics and four IMUs for lower-limb tracking [9]; Joukov et al. proposed a rhythmic extended Kalman filter (R-EKF) based on periodic motion assumptions [10]. More recently, Luke Sy et al. [11] introduced a constrained Kalman filter (CKF) [12] method using only three IMUs (bilateral shanks and pelvis), incorporating zero-velocity updates (ZUPT) [13] and kinematic constraints for pose estimation. Marcard et al. proposed Sparse Inertial Poser (SIP), which performs offline global optimization to align model predictions with IMU measurements [14]. However, such model-based methods exhibit inherent limitations due to oversimplified physical assumptions, such as treating the knee joint as a perfect hinge or assuming a stationary foot phase. In reality, human motion involves complex nonlinear rotations and soft tissue artifacts (STA) [15,16,17]. When subjects perform non-periodic actions (e.g., side-stepping or jumping), these assumptions often break down, leading to significant error accumulation.

To address these limitations, researchers have increasingly turned to data-driven approaches. Early efforts include nearest-neighbor search by Tautges et al. [18], artificial neural networks by Wouda et al. [19], and generalized regression neural networks by Findlow et al. [20]. With the rise of deep learning, end-to-end pose estimation using sparse sensors has achieved notable advances. Huang et al. proposed Deep Inertial Poser (DIP), which reconstructs full-body pose using six IMUs and bidirectional recurrent neural networks (Bi-RNN) [21]; Yi et al. extended this work with TransPose to address global translation estimation [22]; Hernandez et al. utilized a DeepConvLSTM architecture with five sensors for lower-limb kinematic prediction during walking and running [23,24,25]; Zimmermann et al. explored deep convolutional networks for sensor signal processing [26]. Despite their strong modeling capabilities, these methods face two major challenges in real-world applications. First is the simulation-to-reality gap: models such as DIP and TransPose rely heavily on synthetic datasets like AMASS [27] generated from the SMPL model [28]. These simulated IMU signals fail to capture real-world disturbances such as magnetic field distortions, sensor bias drift, and skin motion artifacts caused by muscle contractions [29], resulting in significant performance degradation when deployed on real data. Second is the limitation in modeling long-term temporal dependencies: traditional LSTM or Bi-RNN architectures lack effective global self-attention mechanisms [30], making it difficult to retain critical kinematic phase information in long sequences. This often leads to severe angle drift (with errors reaching 13°–16° on real datasets [22]), which is insufficient for precise clinical evaluation.

To address the limitations of the aforementioned model-based approaches, this paper proposes a high-precision lower-limb kinematic reconstruction system based on a minimalist wearable sensor network and a deep learning architecture. The main contributions of this work are as follows:

1. Minimal hardware configuration: The feasibility of a 3-IMU setup (“bilateral shanks + pelvis”) for lower-limb motion capture is validated. The proposed system acquires essential kinematic information using only three sensors, significantly reducing hardware cost while improving wearing comfort in daily applications.
2. Single IMU temporal feature preprocessing: At the low-level sensor data preparation stage, adaptive modifications are made to the traditional gradient descent attitude algorithm. By attempting channel decoupling of acceleration and magnetic field within a single IMU node, it aims to provide the backend neural network with temporal relative features characterized by smoother waveforms and relatively controlled low-frequency drift, serving as a robust input source for the deep learning model.
3. Data-driven temporal reconstruction: A Transformer-based neural network architecture is innovatively applied to lower-limb pose estimation. Leveraging the powerful self-attention mechanism of deep learning models to capture long-range temporal dependencies, this approach replaces traditional complex biomechanical modeling. It enables end-to-end inference from the observation sequences of three local nodes to the full lower-limb kinematic poses—particularly for uninstrumented segments such as the thighs—thereby effectively avoiding systematic errors introduced by imperfect biomechanical assumptions.

## 2. Materials and Methods

### 2.1. Design of a Distributed Wireless Sensing Hardware System

**Figure 1:**
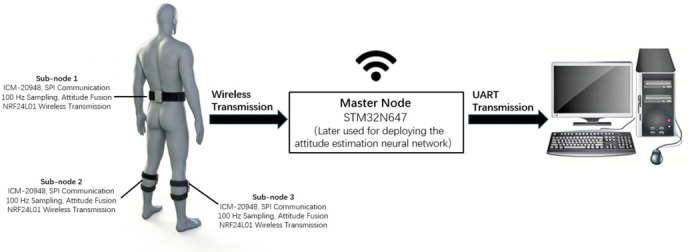
Data transmission diagram of the distributed wireless acquisition system.

The proposed system establishes a wireless inertial sensing network consisting of three distributed sensing nodes and one central master node, as illustrated in Figure 1. Each sensing node is equipped with an ICM-20948 (TDK InvenSense, San Jose, CA, USA) inertial measurement unit (IMU). To ensure real-time performance under high-dynamic motion, all nodes synchronously acquire raw inertial data at a sampling rate of 100 Hz via the SPI communication protocol. After performing preliminary orientation estimation on the onboard microcontroller, each node packages the computed quaternion, along with raw acceleration and angular velocity data, and transmits them to the master node using an NRF24L01 (Nordic Semiconductor, Trondheim, Norway) wireless communication module. Upon receiving the data packets from all sensing nodes, the master node forwards them to a host computer via serial communication for storage. This distributed architecture effectively distributes the computational load and eliminates the physical constraints imposed by wired connections, thereby providing a reliable hardware foundation for capturing natural human motion in unconstrained environments. To ensure the mutual independence of high-frequency data acquisition and computationally intensive inference tasks, a lightweight real-time operating system (RTOS) is deployed on the master node STM32N647 (STMicroelectronics, Geneva, Switzerland), adopting a strict non-blocking multi-task parallel processing architecture. The wireless receiver module (NRF24L01) is configured with the highest priority Direct Memory Access (DMA) mechanism. When the 100 Hz data frames uploaded by the distributed sensing nodes arrive, the hardware directly transfers the data packets into a circular buffer in the memory via DMA. This entire process operates completely independent of the CPU, fundamentally eliminating the risk of data frame loss caused by processor overhead.

### 2.2. Experimental Protocol and Data Collection

**Figure 2:**
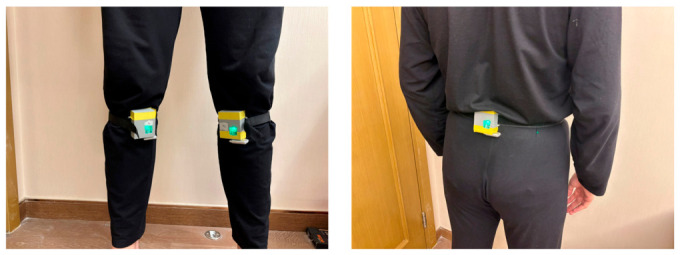
Participant sensor placement diagram.

In terms of sensor placement, this study adopted a minimal 3-IMU layout (“pelvis + bilateral shanks”), as illustrated in Figure 2. The two shank sensors were firmly secured on the medial flat surfaces of both tibiae, where the subcutaneous tissue is relatively thin, effectively reducing vibration noise caused by muscle tremors during movement. The third sensor was fixed at the central posterior position of the pelvis, serving as a global reference for kinematic computation. The experiments were conducted in a laboratory equipped with the NOKOV Motion Capture System MARS2H (NOKOV Science and Technology Co., Ltd., Beijing, China), an 8-camera high-precision optical motion capture system, which was also set to a 100 Hz sampling rate.

Twelve healthy volunteers were recruited as participants, including ten males and two females. Under the raw 100 Hz sampling rate of the wireless sensing network, the single-round data collection duration of the “level walking” task for each participant was 6 min, and that for each of the remaining 6 typical daily movements was 2 min, accumulating a total database of approximately 216 min (about 1,296,000 frames) of continuous lower-limb motion temporal signals. Their basic physical characteristics were as follows: age 24.5 ± 2.1 years, height 172.4 ± 6.8 cm, and weight 60.2 ± 12.5 kg (mean ± std). The participants’ body types ranged from slender to slightly overweight, with significant variation in height, ensuring that the model could learn diverse movement features arising from differences in limb leverage and mass distribution. All participants had no lower-limb motor dysfunction or recent injury history, and they provided informed consent prior to the experiments.

**Figure 3:**
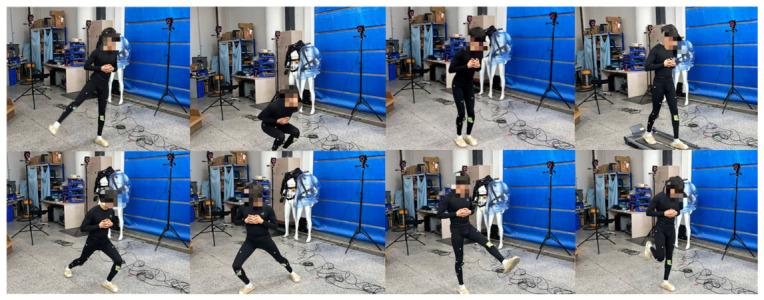
Participant motion collection diagram.

The performed tasks included seven typical daily movements: level walking, squatting, forward and backward lunges, and multidirectional leg lifts, as illustrated in Figure 3. These movements cover the primary sagittal and coronal plane motions of the lower limbs, providing the model with rich samples of non-periodic movement features.

Since the inertial system and the optical motion capture system lack hardware-level synchronization triggers, this study employed an asynchronous alignment strategy based on nearest timestamps. Upon receiving each IMU data packet, the host computer automatically recorded a high-precision Unix real-time timestamp. During offline processing, the IMU timeline was matched with the original motion capture timeline by identifying data frames with the minimal time interval, thereby correcting the initial phase offset between the two systems.

To evaluate the accuracy of the software-based asynchronous alignment strategy under highly dynamic conditions, this study introduces a normalized cross-correlation analysis of attitude quaternions based on temporal waveform similarity. By normalization, the method eliminates signal energy differences and is inherently insensitive to amplitude magnitude. Its core objective is to quantify the phase synchronization between the IMU sensing signals and the ground truth reference labels.

(1) $$R(\tau )=\frac{\sum_{t}\left[\left(Q_{w,\mathrm{IMU}}(t)-{\bar{Q}}_{w,\mathrm{IMU}}\right)\cdot \left(Q_{w,\mathrm{Mocap}}(t+\tau )-{\bar{Q}}_{w,\mathrm{Mocap}}\right)\right]}{\sqrt{\sum_{t}{\left(Q_{w,\mathrm{IMU}}(t)-{\bar{Q}}_{w,\mathrm{IMU}}\right)}^{2}\cdot \sum_{t}{\left(Q_{w,\mathrm{Mocap}}(t)-{\bar{Q}}_{w,\mathrm{Mocap}}\right)}^{2}}}$$

By calculating the sliding cross-correlation coefficient between the real parts of the IMU-derived leg orientation sequence $Q_{w,\mathrm{IMU}}(t)$ and the co-axial rigid body orientation sequence $Q_{w,\mathrm{Mocap}}(t)$ from the MoCap system, the results indicate that the mean optimal time lag at the maximum cross-correlation coefficient is only 4.2 ms. The maximum residual delay is strictly bounded within a single physical sampling period (10 ms). This quantitative result confirms that the minor phase error introduced by the software synchronization strategy is negligible relative to macroscopic lower-limb movements, thereby successfully establishing a high-quality, standardized lower-limb motion dataset with strict temporal alignment.

### 2.3. Adaptive Attitude Decoupling Algorithm

To provide temporal relative features that are easier to learn for the backend deep learning network, the system performs basic data preprocessing at the single sensor node level. Instead of adopting a complex independent classic filter system, this study makes targeted engineering modifications based on the classic gradient descent method. Serving merely as a front-end feature decoupling tool for a single IMU module, this algorithm introduces a dynamic confidence factor to weaken the transient impacts generated by intense movements, thereby ensuring the continuity of input features.

#### 2.3.1. Fundamental Quaternion Kinematics Update

The proposed system utilizes a quaternion $q={\left[q_{0},q_{1},q_{2},q_{3}\right]}^{T}$ to represent the spatial orientation of the sensors. Based on the three-axis angular velocity measurements from the gyroscope, denoted as $\omega^{s}={\left[0,\omega_{x}^{s},\omega_{y}^{s},\omega_{z}^{s}\right]}^{T}$, the differential equation is discretized. The a priori orientation update formula, which relies solely on the integration of the gyroscope data, is given by:

(2) qω,tns=q^est,t−1ns+12(q^est,t−1ns⊗ωs)Δt

Due to the inherent low-frequency drift of the gyroscope, it is necessary to introduce the accelerometer and magnetometer for gradient descent compensation.

#### 2.3.2. Decoupling of Motion Acceleration and Adaptive Derivation of Roll/Pitch Angles

Under ideal quasi-static conditions, the accelerometer measures only gravity. By transforming the normalized gravity vector $g^{n}={\left[0,0,1\right]}^{T}$ from the geographic coordinate system to the sensor coordinate system and subtracting it from the actual accelerometer measurement $a^{s}={\left[a_{x}^{s},a_{y}^{s},a_{z}^{s}\right]}^{T}$, the acceleration error objective function f(qns,as) is defined as:

(3) f(qns,as)=2(q1q3−q0q2)−axs2(q0q1+q2q3)−ays2(0.5−q12−q22)−azs

By taking the partial derivative of this error function, the Jacobian matrix under the acceleration constraint, Ja(qns). From this, the gradient correction direction for acceleration, ∇fa=JaTf(qns,as), can be derived. To decouple the interference of motion acceleration, an acceleration confidence factor $\lambda_{a}$ is introduced. The first independent orientation update formula is given by:

(4) qest1,tns=qω,tns−λaβ∇fa||∇fa||Δt

The system quantifies the intensity of the motion by calculating the standard deviation, $S_{a}$, of the magnitude of *N* consecutive acceleration measurements in real-time:

(5) $$S_{a}=\sqrt{\frac{\sum_{i=1}^{N}{\left(a_{i}^{s}-\bar{a^{s}}\right)}^{2}}{N}}$$

A quasi-static threshold $\zeta_{a}$ is defined: when $S_{a}\leq \zeta_{a}$, $\lambda_{a}$ is set to 1; when $S_{a}>\zeta_{a}$, which indicates the presence of vigorous lower-limb movement, $\lambda_{a}$ is automatically set to 0.01 to significantly suppress the correction from anomalous acceleration. In this study, the gradient descent step size β is set to 0.1, and the sliding window size is chosen as *N* = 20, a length that can sensitively capture the characteristics of lower-limb movements switching from static to dynamic states. The quasi-static threshold is set to $\zeta_{a}=0.01\;g$, which is strictly measured based on the inherent baseline noise variance of the sensor in an ideal static state. When human movement causes the real-time standard deviation to exceed this value, the confidence factor $\lambda_{a}$ immediately drops from 1 to 0.01.

#### 2.3.3. External Magnetic Field Decoupling and Adaptive Derivation of the Yaw Angle

The Earth’s magnetic field has components in both the horizontal and vertical planes. Let the magnetometer measurement be $m^{s}={\left[m_{x}^{s},m_{y}^{s},m_{z}^{s}\right]}^{T}$. Transforming this into the geographic coordinate system yields $m^{n}={\left[0,m_{x}^{n},m_{y}^{n},m_{z}^{n}\right]}^{T}$. To eliminate the effect of magnetic declination, the horizontal magnetic field components are merged towards the geomagnetic North Pole, yielding the local theoretical magnetic field reference vector $b^{n}$:

(6) $$b^{n}={\left[b_{x}^{n},0,b_{z}^{n}\right]}^{T}={\left[\sqrt{{\left(m_{x}^{n}\right)}^{2}+{\left(m_{y}^{n}\right)}^{2}},0,m_{z}^{n}\right]}^{T}$$

By transforming $b^{n}$ to the sensor coordinate system and subtracting it from $m^{s}$, the magnetic field error objective function f(qns,ms) is constructed:

(7) f(qns,ms)=2bxn(0.5−q22−q32)+2bzn(q1q3−q0q2)−mxs2bxn(q1q2−q0q3)+2bzn(q0q1+q2q3)−mys2bxn(q0q2+q1q3)+2bzn(0.5−q12−q22)−mzs

Similarly, by taking the partial derivative with respect to the quaternion variables in this error function, the Jacobian matrix under the magnetic field constraint, Jm(qns). The magnetic field gradient direction, ∇fm=JmTf(qns,ms), is then calculated. Similarly, to resist transient external ferromagnetic interference, a magnetic field confidence factor $\lambda_{m}$ is introduced to perform the second independent correction for the heading angle (yaw):

(8) qest2,tns=qω,tns−λmβ∇fm||∇fm||Δt

The magnitude of the measured magnetic field is defined as $L_{m}$, and the decision threshold is defined as $\zeta_{m}$. When $L_{m}\leq \zeta_{m}$, it is determined that there is no magnetic interference, and $\lambda_{m}$ is set to 1. When $L_{m}>\zeta_{m}$, which indicates the presence of intense magnetic interference, $\lambda_{m}$ is automatically set to 0.01 to significantly suppress the correction from anomalous magnetic fields. Similarly, the confidence factor β is assigned a value of 0.1, and the magnetic field decision threshold is set to $\zeta_{m}=58$. This value is determined based on the static baseline calibration of the local geomagnetic field strength under magnetically undisturbed conditions. In the present experimental environment, the measured geomagnetic field magnitude typically ranged from 57 to 59 μT, and therefore $\zeta_{m}$ was set to 58 μT while allowing a reasonable tolerance margin. It should be noted that $\zeta_{m}$ is not a universal constant. When the system is deployed in a different geographical region or magnetic environment, the local geomagnetic baseline may vary and $\zeta_{m}$ should be recalibrated by estimating the local reference magnetic field magnitude before use. The low-confidence coefficient is fixed at 0.01. This selection is motivated by the need to strongly suppress 99% of erroneous corrections caused by severe magnetic disturbances, ensuring that the system primarily relies on uncontaminated gyroscope integration to maintain short-term accuracy during interference periods. Meanwhile, a weak residual physical feedback is preserved to guarantee smooth convergence of the attitude quaternion once the disturbance subsides and $\lambda_{m}$ returns to 1, thereby avoiding numerical discontinuities or divergence.

#### 2.3.4. Multi-Source Attitude Feature Fusion Output

**Figure 4:**
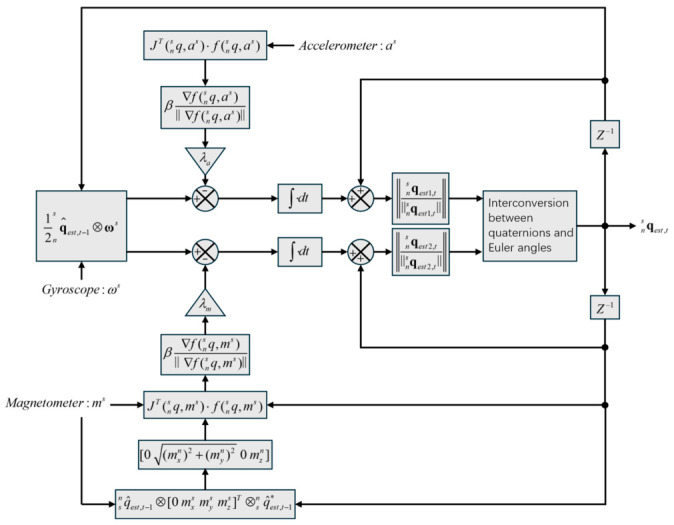
Flowchart of the IMU-based adaptive gradient descent attitude decoupling and fusion algorithm.

After completing the two independent decoupling iterations, to obtain the optimal full spatial orientation representation, $q_{\mathrm{est}1,t}$ is converted into Euler angles to extract the roll angle $\phi_{a}$ and pitch angle $\theta_{a}$, which are effectively immune to motion acceleration. Simultaneously, $q_{\mathrm{est}2,t}$ is converted into Euler angles to extract the yaw angle $\psi_{m}$, which is free from magnetic field interference. Finally, the recombined Euler angle vector ${\left[\phi_{a},\theta_{a},\psi_{m}\right]}^{T}$ is converted back into the final orientation quaternion qest,tns. The workflow of the proposed algorithm is illustrated in Figure 4.

Through the underlying engineering-oriented preprocessing introduced by the proposed improved algorithm, the raw temporal features generated by a single IMU are able to suppress high-frequency impulsive noise to a certain extent, thereby providing the subsequent Transformer network with temporally stable feature inputs exhibiting relatively consistent geometric characteristics. It should be noted that this front-end fine-tuning strategy is primarily designed for data cleansing within the present processing pipeline and does not inherently possess the adaptive optimality of conventional sensor fusion filters, such as the Madgwick Filter or Mahony Filter.

### 2.4. Multimodal Data Spatial Alignment

After low-level processing, the host system receives quaternion, acceleration, and angular velocity data from the three nodes located at the bilateral shanks and the pelvis. To enable the neural network to better learn human kinematic constraints, it is necessary to perform a strict physical spatial transformation between the input features and the ground truth provided by the optical motion capture (Mocap) system.

#### 2.4.1. Input Data Spatial Transformation and Increment Extraction

The originally calculated IMU quaternions are relative to the geographic coordinate system (world frame). To extract the relative motion features between the lower-limb joints, the quaternions ($q_{L},q_{R}$), accelerations $\left(a_{L},a_{R}\right)$, and angular velocities $\left(\omega_{L},\omega_{R}\right)$ of the left and right shanks are first transformed into the local coordinate system relative to the pelvis $\left(q_{P}\right)$. Taking the left shank as an example, its relative orientation quaternion $q_{L\_\mathrm{rel}}$ and relative acceleration $a_{L\_\mathrm{rel}}$ with respect to the pelvis are calculated as follows:

(9) $$q_{L\_\mathrm{rel},t}={\left(q_{P,t}\right)}^{-1}⊗q_{L,t}$$

(10) $$a_{L\_\mathrm{rel},t}=R({\left(q_{P,t}\right)}^{-1})\cdot a_{L,t}$$

Furthermore, due to the inevitable mounting deviations in the initial placement of the sensors across different subjects, this study required all subjects to adopt a strictly vertical standing posture as the initial pose during data collection. Based on this, the network does not need to learn the absolute mounting orientations, but rather the variations in orientation. The transformed relative quaternion for each frame (as well as the raw quaternion of the pelvis) is multiplied by the inverse of the initial frame’s quaternion (at the standing moment t = 0):

(11) $$\Delta q_{L\_\mathrm{rel},t}={\left(q_{L\_\mathrm{rel},0}\right)}^{-1}⊗q_{L\_\mathrm{rel},t}$$

After the aforementioned processing, the input data are transformed into incremental quaternion features that purely reflect human motion changes, significantly reducing the static errors caused by sensor placement displacement.

#### 2.4.2. Hierarchical Processing of Ground Truth

The ground truth provided by the Mocap system consists of relative quaternions for five key segments: the orientation of the pelvis with respect to the global coordinate system, the orientations of the left and right thighs relative to the pelvis, and the orientations of the left and right shanks relative to their corresponding thighs.

To ensure consistency with the incremental representation adopted for the input features, an initial-frame normalization is applied to all five quaternion sequences in the Mocap ground truth. Specifically, each sequence is aligned with its first frame, so that the resulting representations capture only the temporal variations in motion rather than absolute orientations.

(12) $$\Delta q_{\mathrm{GT},t}^{i}={\left(q_{\mathrm{GT},0}^{i}\right)}^{-1}⊗q_{\mathrm{GT},t}^{i}\;(i\in \{\mathrm{Hip},L\_\mathrm{Thigh},R\_\mathrm{Thigh},L\_\mathrm{Shank},R\_\mathrm{Shank}\})$$

This formulation ensures that the network’s regression objective is exclusively focused on the dynamic variations in joint angles.

### 2.5. Pure Self-Attention-Based Kinematic Reconstruction Network

After defining the incremental input features and the hierarchical relative ground truth, this study designs a cascaded two-layer Transformer architecture tailored to the characteristics of IMU time-series signals.

#### 2.5.1. Network Architecture Design

**Figure 5:**
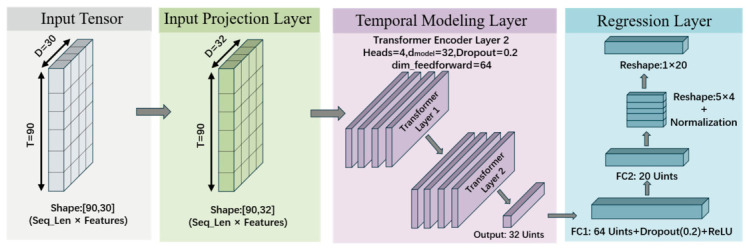
Architecture diagram of the cascaded two-layer transformer network.

The proposed lower-limb kinematics reconstruction network employs a pure self-attention mechanism (Pure Transformer) architecture, which avoids the cumulative errors associated with complex physical models. The network primarily consists of three cascaded core modules: input feature projection, temporal modeling, and kinematic regression. Its complete architecture is illustrated in Figure 5.

1. Linear Feature Projection Layer: The input to the network is a sliding window sequence with a time step T = 90, corresponding to a temporal span of 0.9 s under a sampling frequency of 100 Hz. Through empirical tuning, this window length effectively captures the key phase transitions within a complete human motion cycle while avoiding excessive edge inference latency. At the same time, it provides sufficient long-range contextual temporal information for the self-attention mechanism, and a feature dimension D = 30, denoted as $X\in {\mathbb{R}}^{90\times C_{\mathrm{in}}}$ (where $C_{\mathrm{in}}$ is the number of channels after concatenating $\Delta q,a_{\mathrm{rel}},\omega_{\mathrm{rel}}$ from all nodes). To accommodate the computational requirements of the self-attention mechanism, the sequence first passes through a linear projection layer, which maps the raw sensor signals from the 30-dimensional space to a high-dimensional feature space with $d_{\mathrm{model}}=32$, outputting a tensor of shape [90, 32]. Compared with traditional convolutional feature extraction, linear projection can more directly and lossless preserve the transient variation patterns of the original physical signals in the temporal dimension, providing a unified feature embedding for subsequent global modeling.
2. Core Temporal Modeling Layer: After the linear feature projection, since the Transformer inherently lacks the ability to process sequence order, the system first injects sinusoidal positional encoding into the feature tensor to preserve the sequential relationship of the gait data. Subsequently, the data enters the temporal modeling module, which is stacked with two Transformer Encoder layers. The specific hyperparameters are set as follows: the multi-head attention mechanism contains 4 attention heads (Heads = 4), the model dimension $d_{\mathrm{model}}=32$, the feed-forward neural network dimension dim_feedforward=64, and a dropout rate of 0.2 is applied to prevent overfitting. By calculating the global correlation weights among all frames within the sliding window, the self-attention mechanism can automatically focus on the historical motion patterns most relevant to the current posture inference, thereby effectively overcoming the angular drift problem in large-range nonlinear movements.
  Experimental results indicate that increasing the model dimensionality yields diminishing marginal improvements in reconstruction accuracy, while simultaneously causing an exponential growth in the static memory consumption of the microcontroller. Therefore, the current lightweight configuration with $d_{\mathrm{model}}=32$ and Heads=4 represents an optimal trade-off between reconstruction accuracy and edge hardware overhead. The configuration is designed to meet the stringent Flash and RAM constraints of resource-limited embedded edge platforms (STM32N6), ensuring that the quantized model can be fully accommodated within the on-chip memory of the microcontroller, thereby enabling real-time edge inference with a latency of approximately 17 ms.
3. Kinematic Regression and Normalization Output Layer: The hidden state sequence output by the Transformer is first subjected to Global Average Pooling (GAP) for dimensionality reduction along the temporal dimension, extracting a 32-dimensional vector that encapsulates the global motion features. Subsequently, this vector is fed into a Multi-Layer Perceptron (MLP) consisting of fully connected layers for posture regression. The MLP comprises two fully connected layers: the first layer (FC1) expands the dimensionality to 64 and is coupled with a ReLU activation function and Dropout (0.2) to enhance the nonlinear representation capability; the second layer (FC2) compresses the features to 20 dimensions. To satisfy the physical definition of quaternions, this 20-dimensional vector is first reshaped into a 5 × 4 matrix (representing the relative posture variations of the 5 key segments of the lower limbs) and undergoes L2 norm normalization. This ensures that the prediction result for each segment maintains strict mathematical rationality within the SO(3) rotation space. Finally, the output is reshaped back into a 1 × 20 format, serving as the final estimated output of the lower-limb posture at the current time step.

#### 2.5.2. Training Strategy and Dataset Partitioning

To evaluate the network’s generalization capability on unseen individuals, a rigorous dataset splitting strategy was adopted: the complete motion data from 8 subjects were allocated as the training set, 2 subjects as the validation set for early stopping and hyperparameter tuning, and the remaining 2 subjects were reserved as an independent test set.

For data segmentation, a sliding window with a fixed length of 90 frames was employed to partition the temporal sequences. To ensure full reproducibility of the proposed study, all critical hyperparameters were strictly specified and fixed during the network training stage. A global random seed of 42 was enforced throughout the training process. The model was optimized using the Adam optimizer, and the optimal initial learning rate was determined as 0.000342 through single-objective hyperparameter optimization, together with the introduction of L2 regularization-based weight decay. The maximum number of training epochs was set to 100.

Meanwhile, to mitigate overfitting in the deep temporal network, an Early Stopping mechanism based on the validation loss was adopted. The monitored metric was the geodesic geometric loss on the validation set, with a patience value of 10 epochs. If the validation loss failed to achieve a new minimum within 10 consecutive epochs, the training procedure was automatically terminated early, and the model weights corresponding to the best validation performance were restored and preserved.

In addition, before the data stream was fed into the network, two forms of online regularization were introduced to reduce the simulation-to-reality gap. Firstly, Gaussian random noise with a predefined standard deviation of 0.01 was superimposed on the original incremental input features. Second, a random channel masking strategy with a probability of 5% was employed. Specifically, during training, certain physical channel elements within the input matrix were randomly forced to zero with a probability of 5%, thereby compelling the self-attention mechanism to autonomously recover the underlying kinematic constraints from incomplete temporal manifolds.

During the training process, considering the inherent double-cover property of quaternions when representing spatial rotations, the traditional Euclidean Mean Squared Error (MSE) was discarded. Instead, a specific Quaternion Distance Loss was designed and utilized. This loss function quantifies the orientation deviation by calculating the absolute value of the dot product between the predicted quaternion and the ground truth (MoCap), defined as $L=1-|\langle q,\hat{q}\rangle |$. This approach elegantly circumvents the training instability issues caused by the antipodal sign ambiguity (i.e., *q* and −*q* representing the same rotation). The Adam optimizer was employed for parameter updating. Ultimately, the model weights that achieved the optimal performance on the validation set were saved for subsequent quantization and deployment on the embedded edge device.

### 2.6. Model Quantization and Embedded Deployment Evaluation Based on CubeAI

To enable unconstrained daily gait monitoring, edge deployment of deep learning models is essential. In this system, the STM32Cube.AI v10.0.0 (STMicroelectronics, Geneva, Switzerland) toolchain provided by STM32CubeMX v6.15.0-RC4 (STMicroelectronics, Geneva, Switzerland) was employed to convert the floating-point Transformer model trained in PyTorch 2.7.1+cu128 (Meta Platforms, Menlo Park, CA, USA) into a highly optimized C-based inference library, which was seamlessly integrated into the STM32N647 master node firmware.

During deployment, network weights were quantized and operator fusion was applied to accommodate the memory constraints of the microcontroller. By leveraging the multi-axis matrix parallel computing architecture of the built-in neural processing unit (NPU) on the STM32N647, the microcontroller can efficiently execute the matrix multiplication operations inherent in the multi-head self-attention mechanism of Transformer architectures.

Performance evaluation of the deployed model demonstrated extremely low memory usage on the STM32N647 platform. Flash memory consumption was only 196.89 KB (which can be easily accommodated in the external Quad-SPI Flash), and RAM usage was 240.09 KB (representing a tiny fraction of the on-chip 6.5 MB SRAM bandwidth, ensuring massive computational headroom). The model requires approximately 31.03 million multiply–accumulate operations per inference. To ensure data integrity in a multi-tasking environment, the system adopts a decoupled sliding-window stride with a parallel pipeline strategy. Although the raw data acquisition and reception frequency of the distributed wireless sensing nodes is maintained at 100 Hz (i.e., a 10 ms sampling period), the deployed Transformer model operates at a lower output and inference frequency of 50 Hz (i.e., a 20 ms inference period).

Specifically, whenever the main controller receives two new frames of data via the underlying DMA (accumulating 20 ms of samples), the RTOS updates the sliding-window input tensor containing 90 consecutive frames and triggers the model inference task in an event-driven manner. Benefiting from the hardware acceleration provided by the STM32N647 NPU, the inference time for a single 90-frame sliding window kinematic prediction is approximately 17 ms.

Since the inference latency is strictly shorter than the 20 ms triggering period, the system maintains sufficient computational headroom in each processing cycle. This enables smooth pipelined parallel execution without blocking high-frequency data acquisition tasks. The resulting millisecond-level ultra-low latency demonstrates that the system can operate independently of an offline host computer, fully supporting real-time edge inference for human posture perception and motion intention estimation.

### 2.7. Quantitative Evaluation Metrics

To quantitatively evaluate the tracking accuracy of the CNN–Transformer network for the lower-limb joints (particularly the uninstrumented thigh segments), the Mean Absolute Error (MAE) was selected as the primary evaluation metric. Let *N* represent the total number of frames in the testing sequence, $\theta_{k}$ denote the ground truth joint angle (or Euler angle) measured by the optical motion capture system at the *k*-th frame, and $\theta_{k}$ denote the corresponding angle predicted by the proposed network. The MAE, which quantifies the absolute deviation between the predicted values and the ground truth, is calculated as follows:

(13) $$\mathrm{MAE}=\frac{1}{N}\sum_{k=1}^{N}|{\hat{\theta }}_{k}-\theta_{k}|$$

The Mean Absolute Error (MAE) is employed to quantify the absolute magnitude deviation between the predicted values and the ground truth. However, as a scalar absolute-error metric, MAE is incapable of evaluating the morphological similarity and phase synchronization of temporal signals. Therefore, this study further introduces the Pearson Correlation Coefficient (PCC) as a complementary quantitative metric for assessing temporal consistency. PCC is used to characterize the degree of linear correlation between two time series in terms of motion trends, and its formulation is given as follows:

(14) $$\mathrm{PCC}=\frac{\sum_{k=1}^{N}\left(y_{k}-\bar{y}\right)\left({\hat{y}}_{k}-\bar{\hat{y}}\right)}{\sqrt{\sum_{k=1}^{N}{\left(y_{k}-\bar{y}\right)}^{2}\cdot \sum_{k=1}^{N}{\left({\hat{y}}_{k}-\bar{\hat{y}}\right)}^{2}}}$$

where $\overset{ˉ}{y}$ and $\overset{ˉ}{\hat{y}}$ denote the statistical means of the ground truth joint angle sequence and the model-predicted joint angle sequence, respectively. The value range of PCC is $\left[-1,\;1\right]$. A PCC value closer to 1 indicates a higher degree of phase synchronization and stronger waveform similarity between the predicted trajectory and the reference optical motion-capture trajectory along the temporal axis.

## 3. Results

### 3.1. Model Training and Convergence Analysis

**Figure 6:**
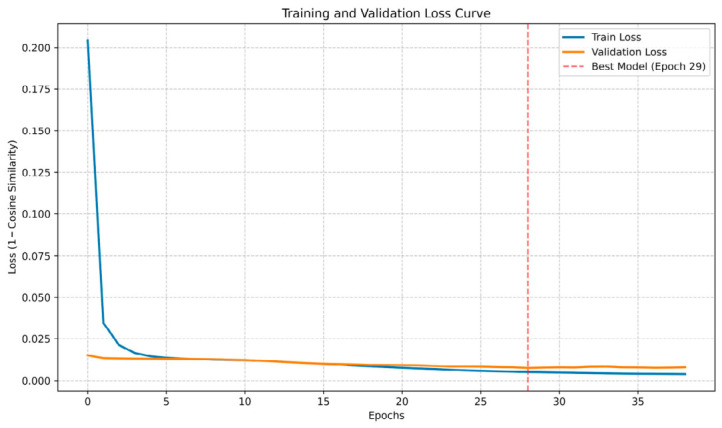
Training and validation learning curves of the kinematic reconstruction network.

To quantitatively characterize the fitting behavior and generalization robustness of the proposed lower-limb kinematic reconstruction network throughout the entire training process, this section independently evaluates the convergence characteristics of the model. Figure 6 presents the learning curves of the network on both the training set and the independent validation set. The core monitored metric is the quaternion absolute cosine distance loss $\left(1\;-\;\mathrm{Cosine}\;\mathrm{Similarity}\right)$, which is employed to evaluate the similarity deviation within the three-dimensional attitude space.

As illustrated by the quantitative trajectories in Figure 6, both loss curves exhibit highly consistent convergence behavior and remarkable stability. Specifically, the training loss decreases monotonically in a cliff-like manner from approximately 0.204 at Epoch 0 and rapidly drops to around 0.035 within the first training epoch. This phenomenon indicates that the pure Transformer architecture is capable of capturing the spatial kinematic features embedded in the distributed IMU temporal sequences with extremely high sensitivity and efficiency. As the training process proceeds, the training loss continues to decrease smoothly and gradually converges to approximately 0.004.

Meanwhile, the validation loss demonstrates exceptionally high data adaptability. Its initial value already remains at a relatively low level of approximately 0.015, and throughout the entire 38-epoch training trajectory, the curve maintains highly stable convergence characteristics. During the later training stage, the validation loss steadily stabilizes around a low convergence plateau near 0.008. After smoothly converging around Epoch 4, the two loss curves remain closely aligned over a long training interval, exhibiting no signs of divergence, rebound, or overfitting behavior between the training and validation losses.

Ultimately, according to the adopted weight optimization strategy, the training procedure triggered early stopping near Epoch 29, where the validation loss reached its minimum convergence plateau, and the globally optimal model weights were automatically preserved and locked, as indicated by the red dashed line in Figure 6.

### 3.2. Kinematic Posture Reconstruction Results and Accuracy Analysis

#### 3.2.1. Qualitative Analysis of Temporal Tracking Trajectories

**Figure 7:**
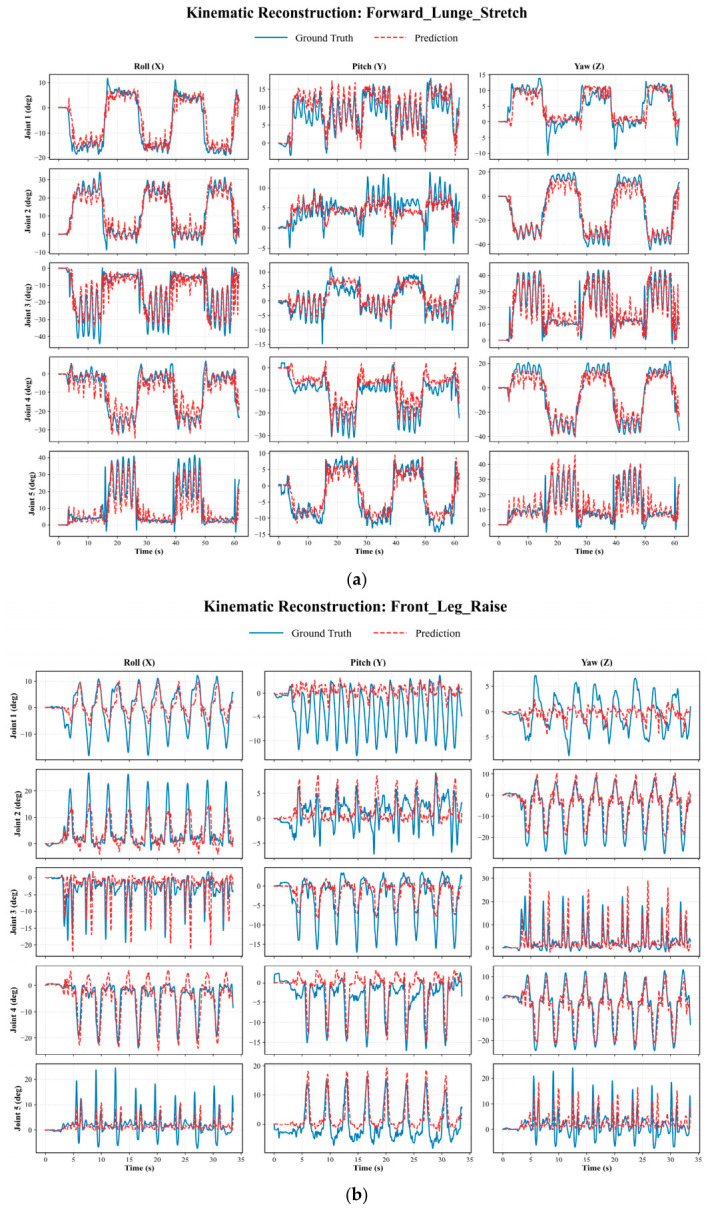

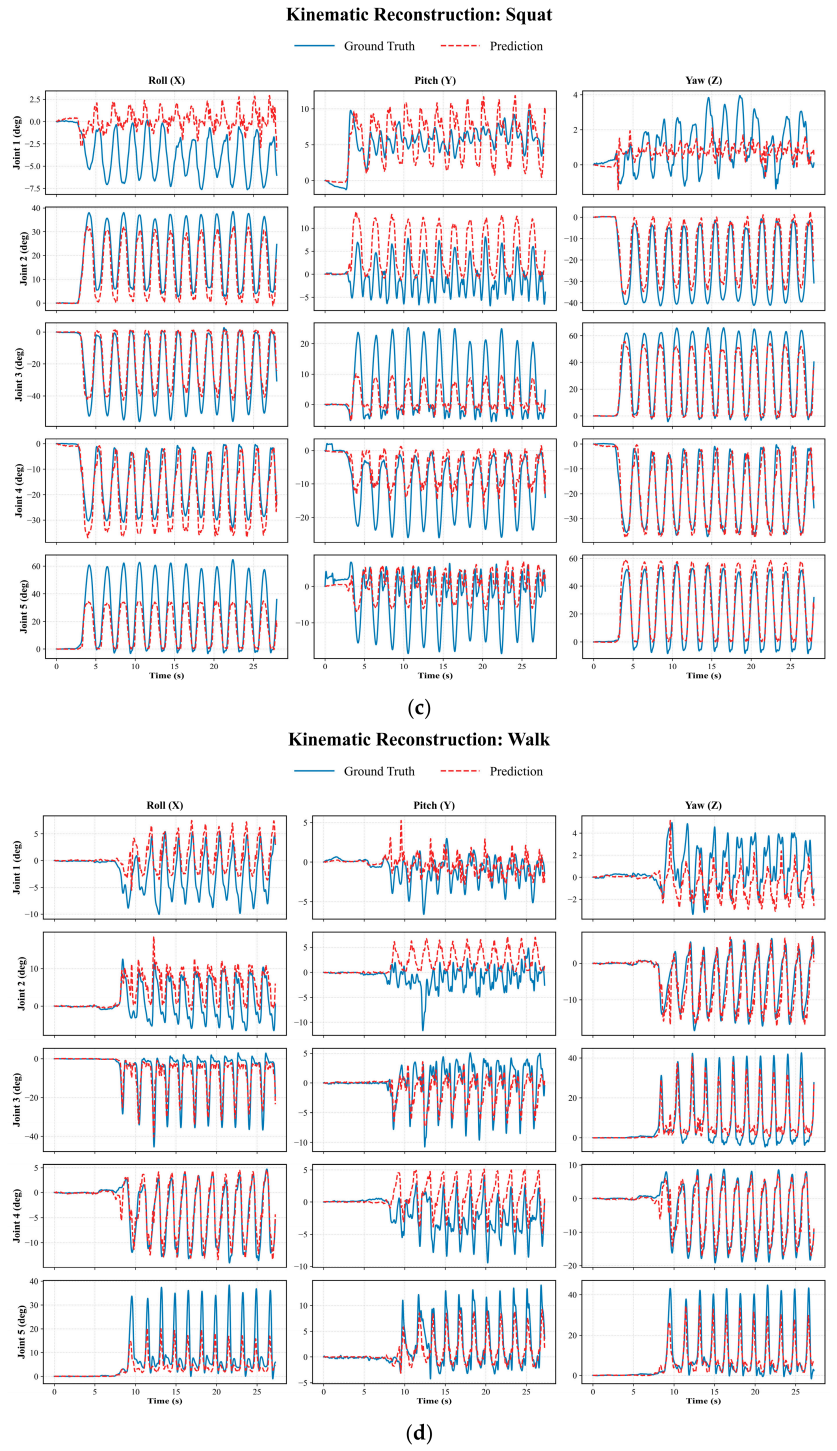
Comparison between reconstructed lower-limb kinematic trajectories and ground truth measurements during representative daily activities. (Red curves: predict/model predictions; blue curves: target/optical motion-capture ground truth.) Subfigures (**a**–**d**) correspond to: (**a**) forward lunge stretch; (**b**) front leg raise; (**c**) squat; and (**d**) walking. The numerical labels in the figure denote the lower-limb segments as follows: 1—pelvis, 2—left thigh, 3—left shank, 4—right thigh, and 5—right shank.

In Figure 7, labels 1–5 correspond to the pelvis, left thigh, left shank, right thigh, and right shank segments, respectively. From the kinematic tracking curves, it can be observed that, for the level-ground walking task, the proposed network is capable of effectively capturing the phase transitions and temporal frequency characteristics of the gait cycle. The predicted trajectories exhibit globally consistent fluctuation patterns with the motion-capture ground truth along the temporal axis, without showing any obvious phase lag in the identification of conventional gait features. Nevertheless, certain limitations still exist in the reconstruction of local motion amplitudes. For example, the left thigh segment (Segment 2) exhibits an observable baseline shift error in the pitch angle, while the motion peaks of the right shank segment (Segment 5) in the roll angle are noticeably underestimated.

For movements involving large lower-limb ranges of motion (ROM), such as squatting, lunge stretching, and forward leg raising, the reconstructed trajectories indicate that the system is able to recover the macroscopic kinematic trends along the primary anatomical motion axes of most joints. Even at the lowest squatting posture or the extreme lunge position, the model still maintains relatively clear temporal tracking of large-amplitude motion patterns. However, constrained by the observation boundaries of the minimal wireless sensor network and the physical influence of soft tissue artifacts (STA), the reconstruction accuracy of certain secondary motion axes or segments without directly attached sensors is inevitably degraded. For instance, during the squatting task, the pitch angle of the left thigh segment (Segment 2) also exhibits a pronounced structural offset. These observations indicate that, although the proposed lightweight data-driven architecture is capable of maintaining globally reliable temporal motion trends, certain limitations still remain in the precise reconstruction of local absolute motion amplitudes.

**Figure 8:**
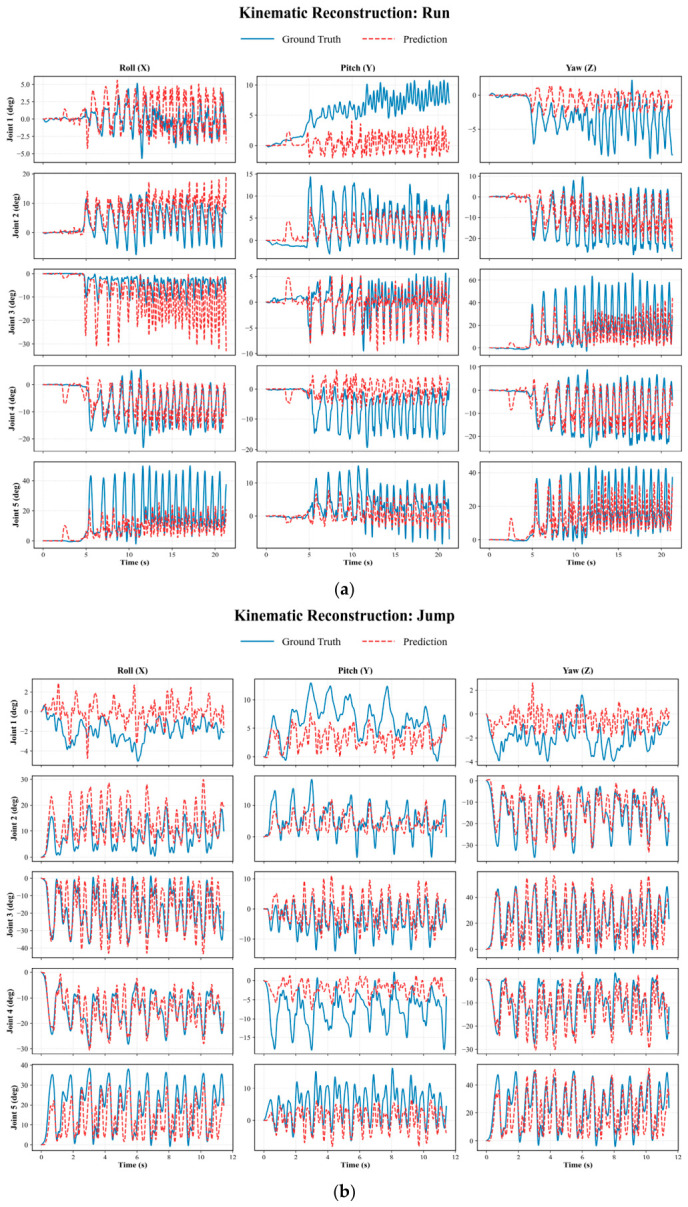
Lower-limb kinematic reconstruction trajectories during highly dynamic activities (running and jumping). (Red curves: predict/model predictions; blue curves: target/optical motion-capture ground truth.) Subfigures (**a**,**b**) correspond to: (**a**) running; and (**b**) jumping. The numerical labels in the figure denote the lower-limb segments as follows: 1—pelvis, 2—left thigh, 3—left shank, 4—right thigh, and 5—right shank.

To further investigate the performance boundaries of the proposed system and evaluate the generalization capability of the model under extreme motion conditions, additional datasets involving highly dynamic activities, including running and bilateral vertical jumping, were collected from the participants. The qualitative reconstruction results are illustrated in Figure 8a,b.

Experimental results demonstrate that, under conditions involving high-frequency impacts and severe transient acceleration disturbances, the reconstruction accuracy of the proposed system decreases noticeably compared with periodic activities such as level-ground walking. This degradation primarily arises because, during running and jumping, substantial impact forces are transmitted throughout the lower limbs, leading to intense muscle oscillations and skin displacement, thereby significantly amplifying the influence of soft tissue artifacts (STA).

In addition, the aggressive non-stationary accelerations encountered during highly dynamic movements frequently exceed the quasi-static thresholds assumed by the front-end decoupling algorithm. Consequently, during certain impact phases, the algorithm excessively suppresses the accelerometer correction gradients, forcing the system to rely predominantly on gyroscope integration over short time intervals, which further introduces cumulative angular drift.

These experimental observations realistically and scientifically reveal the accuracy limitations of the proposed minimal wearable system in practical application scenarios. Moreover, they provide a clear direction for future improvements, particularly through the incorporation of biomechanical constraint terms to suppress non-stationary axial divergence under highly dynamic conditions.

**Figure 9:**
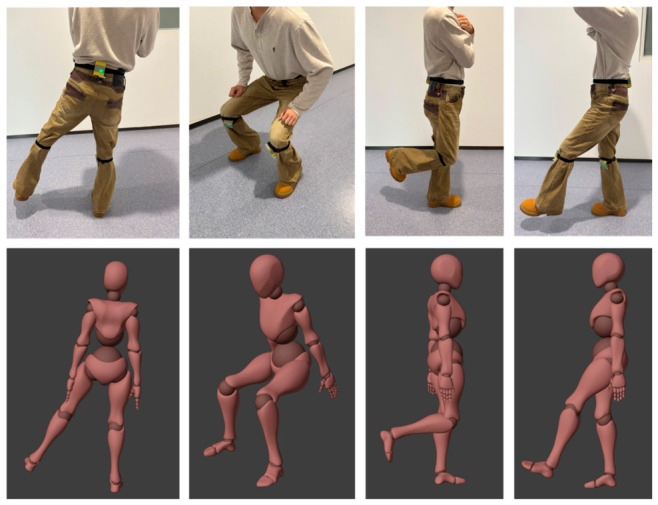
Comparison of typical lower-limb movements with 3D reconstructed models. Four actions are displayed: left leg lift, deep squat, backward leg lift, and walking. The top row shows the real-world action photographs, and the bottom row presents the corresponding 3D skeleton reconstruction models.

Additionally, we conducted an online performance demonstration by equipping the IMUs on the tibiae of both shanks and the pelvis of a single participant. The participant performed four common lower-limb movements, including left and right leg raises, squats, backward leg lifts, and forward leg lifts, as shown in Figure 9. The results indicate that the predicted motions closely match the actual movements, demonstrating a satisfactory fitting performance.

#### 3.2.2. Quantitative Analysis of Joint Reconstruction Accuracy

Table 1 presents detailed error statistics for the system’s inference on the independent test set. Among all movement categories, level walking achieved the highest prediction accuracy and the most stable performance. For this periodic motion, the mean absolute error (MAE) of all lower-limb joints along the three rotational axes was strictly maintained below 4° (with the maximum observed error being only 3.84° for the right shank Roll angle). Notably, reconstruction accuracy in the sagittal plane (Pitch) was particularly high, demonstrating the system’s strong capability to track conventional gait features.

For movements with higher degrees of freedom and more complex joint loading, such as the lunge stretch and left/right leg raises, the hip joint undergoes significant relative rotations in the frontal plane (corresponding to Roll) and transverse plane (corresponding to Yaw), which greatly increases the difficulty of nonlinear mapping. Nevertheless, the Transformer-based model developed in this study still achieved highly competitive results: for the lunge stretch and leg raise tasks, the maximum MAE of all joint angles did not exceed 5°.

It is worth noting that during the squat movement, a relatively high error (9.94°) was observed in the right shank roll angle. This is mainly attributed to the fact that, at the lowest squat position, the gastrocnemius muscle at the posterior side of the shank undergoes substantial compression and is pushed anteriorly, causing slight physical slippage and deflection of the IMU sensor mounted on the medial tibial plane. This introduces pronounced soft tissue artefacts (STA). In addition, the subject exhibits noticeable individual kinematic variations under deep squat conditions. Due to differences in biomechanical habits during daily walking and load-bearing activities among participants in the test set, subject-specific non-axial compensatory motions and local muscle tremors occur when reaching the deepest squat posture. Such inter-individual variability in locomotion and motor habits leads to a distribution shift in the data, which significantly increases the difficulty of modeling this nonlinear axial mapping.

Nevertheless, the overall posture trajectory was still accurately reconstructed, and the correct kinematic trends were well preserved.

Table 2 presents the Pearson correlation coefficient (PCC) statistics of the system across different motion patterns on an independent test set. Overall, the system demonstrates strong trend consistency on anatomical axes with large motion amplitudes and pronounced signal characteristics. For instance, in flat-ground walking, certain axes such as the yaw angles of the thigh and shank achieve PCC values above 0.89, indicating a strong linear correlation between predicted and reference waveforms.

It is noteworthy that the PCC metric shows a significant drop on certain movement axes for specific actions. According to the statistics in Table 2, the most affected typical scenarios include: the pelvis Yaw axis during the Squat task (PCC = 0.048), the left thigh Pitch axis during level walking (Walk) (PCC = 0.032), and the left shank Roll axis during the Side Leg Raise task (PCC = 0.011).

In these scenarios, the corresponding anatomical axes exhibit extremely limited range of motion (ROM). Taking the Squat as an example, the motion primarily occurs in the sagittal plane (Pitch axis), while the pelvis experiences only minimal lateral rotation (Yaw axis), with true-angle fluctuations within ±2°. Under such conditions, the raw physical signals captured by the sensors are easily overwhelmed by baseline noise and soft tissue artifacts, resulting in a very low signal-to-noise ratio. Since the neural network is trained end-to-end to minimize the global quaternion distance loss, the self-attention mechanism of the pure Transformer automatically suppresses the weights of these noisy, low-amplitude channels. Consequently, the regression layer tends to treat them as nearly static constants, mathematically leading to low PCC values due to the misalignment with mocap spurious signals.

Combined with the absolute error statistics in Table 1, although the waveform correlation for these axes is low due to micro-oscillations, their mean absolute errors (MAE) remain strictly below 5° (e.g., Squat pelvis Yaw = 1.16°, Side Leg Raise left shank Roll = 3.12°). This confirms that the model does not produce divergent angles and that the overall pose reconstruction maintains high credibility in absolute magnitude.

### 3.3. Comparison with State-of-the-Art Methods

To further evaluate the overall performance of the proposed system, a comparative analysis was conducted against several representative methods reported in recent literature (as shown in Table 3). It should be noted that, due to differences in dataset characteristics, sensor configurations, and task setups across studies, a strictly controlled comparison is not feasible. However, most existing works report results under the “walking” condition. Therefore, to ensure a relatively comparable evaluation, all reconstruction errors listed in Table 3 are reported under level-ground walking.

Under this benchmark, the conventional kinematic-constrained Kalman filter (CKF) method, despite using an identical three-sensor configuration (pelvis and bilateral shanks), yields a relatively high reconstruction error of 16.1°, indicating certain limitations in achieving high-precision motion reconstruction.

It is worth noting that the TIP method (with an average error of 12.19°) employs up to six IMUs and additionally estimates upper-body posture. As such, it is not strictly equivalent to lower-limb-only approaches, including the proposed method. Furthermore, the TIP model is trained on an idealized physics-based simulation dataset and evaluated on real-world data. In practical scenarios, IMU measurements are inevitably affected by sensor bias drift, random walk noise, and soft tissue artefacts induced by muscle vibrations during dynamic motion. These complex nonlinear disturbances are difficult to faithfully reproduce in simulation environments. The resulting sim-to-real domain gap limits the model’s generalization capability, leading to relatively higher errors in real-world applications.

In contrast, the proposed system is trained end-to-end using real wearable data, enabling the pure Transformer-based architecture to directly learn and adapt to the noise characteristics and kinematic properties of actual hardware. As a result, the method achieves a mean reconstruction error of 2.41° in level-ground walking tasks. Compared with the DeepConvLSTM method, which uses five IMUs and reports an average error of 3.6°, the proposed approach achieves competitive numerical accuracy while relying on only three IMUs, thereby reducing sensor count and wearing complexity.

Overall, these results suggest that integrating a minimal sensor configuration with a self-attention-based deep learning architecture is a promising and effective approach for robust lower-limb kinematic reconstruction in real-world environments.

### 3.4. Ablation Study

To further validate the effectiveness and necessity of the proposed low-level adaptive decoupling strategy and the Transformer-based architecture, a series of ablation studies were conducted on a unified test set using two representative movements: walking and squatting.

The proposed system adopts a cascaded Transformer architecture. To evaluate its superiority, three baseline models were constructed for comparison:

1. CNN–Transformer: A 1D convolutional neural network (1D-CNN) feature extraction layer is introduced before the two-layer Transformer.
2. CNN–LSTM: The front-end 1D-CNN is retained, while the two-layer Transformer is replaced with a long short-term memory (LSTM) network.
3. LSTM: The Transformer is entirely replaced by a two-layer LSTM network.

All comparative models were trained on the same dataset using identical hyperparameters, and their average reconstruction errors were evaluated on the test set.

**Figure 10:**
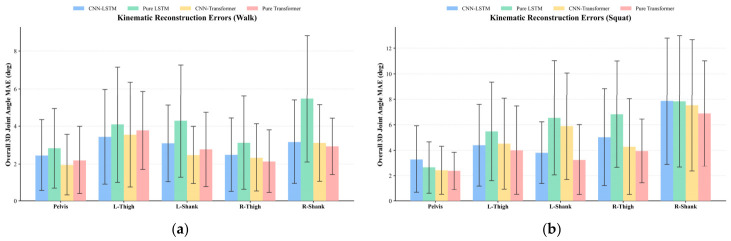
Comparison of kinematic reconstruction errors among different architectures under two typical motion modes. The bar charts demonstrate the overall 3D joint angle mean absolute error (MAE) for the hip, thigh, and shank. The error bars represent the standard deviations. (**a**) Error distribution during the level walking (Walk) task; (**b**) error distribution during the squatting (Squat) task.

First, when examining the capability of different deep learning architectures in extracting kinematic features, the error distribution plots (Figure 10) reveal a clear performance divergence. As illustrated in Figure 10a,b, for both representative movement patterns—walking and squatting—the Transformer-based models (pure Transformer and CNN–Transformer) consistently outperform the LSTM-based counterparts (pure LSTM and CNN–LSTM) in terms of overall three-dimensional pose estimation accuracy across all lower-limb joints.

Notably, the pure Transformer achieves mean absolute error (MAE) values and standard deviations (represented by error bars, reflecting prediction stability) that are highly consistent with those of the more complex CNN–Transformer hybrid model across all joints. This further demonstrates that the pure Transformer can achieve state-of-the-art performance without relying on additional convolutional feature extraction modules.

Furthermore, the detailed quantitative results (as shown in Table 4 and Table 5) further corroborate the aforementioned observations. Specifically, the pure Transformer exhibits nearly identical performance to the CNN–Transformer across all lower-limb segments (pelvis, thighs, and shanks) and all three rotational degrees of freedom (Roll, Pitch, and Yaw). For example, in predicting the pelvis Pitch angle during level walking, the pure Transformer achieves an error of 1.09° ± 1.02°, slightly outperforming the CNN–Transformer (1.11° ± 1.16°). For the squat movement, which involves larger motion amplitudes and stronger nonlinear disturbances, the performance gap between the two models remains within approximately 0.5° for most joints.

These findings strongly demonstrate that the combination of linear projection layers and multi-head self-attention provides powerful global feature representation capabilities. Even without convolutional layers for local receptive field modeling, the pure attention mechanism can directly and accurately capture the nonlinear kinematic mappings embedded in high-frequency IMU signals.

## 4. Discussion

### 4.1. Trade-Off Between Minimal Sensor Configuration and Kinematic Reconstruction Accuracy

The primary objective of this study is to address the “laboratory dependency” limitation of traditional optical motion capture (OMC) systems, as well as the increased wearing complexity caused by redundant sensor configurations in existing wearable solutions. Experimental results demonstrate that the proposed minimal sensor setup—comprising two shank IMUs and one pelvis IMU—combined with a pure Transformer architecture, enables accurate full lower-limb kinematic reconstruction.

Taking the representative level-ground walking task as an example, the proposed system achieves a mean absolute error (MAE) of 2.41° in joint angle estimation. Compared with the conventional kinematic-constrained Kalman filter (CKF) method using the same three-sensor configuration (16.1° ± 3.2°), the proposed approach yields a substantial improvement in accuracy. This performance gain can be attributed to the superior ability of data-driven models to handle complex nonlinear disturbances. Traditional physics-based models rely heavily on idealized assumptions (e.g., modeling the knee joint as a perfect hinge), making them highly sensitive to soft tissue artefacts (STA) and non-periodic motion. In contrast, the Transformer-based model leverages a multi-head self-attention mechanism to effectively capture long-range temporal dependencies, thereby maintaining strong robustness in large-amplitude and complex movements such as squatting and lunging.

At the same time, it should be acknowledged that, due to the lack of a fully unified open benchmark in the field of wearable motion capture, such cross-study comparisons are subject to inherent limitations at a macro level. The compared methods differ in dataset characteristics (synthetic versus real), reconstruction scope (full-body versus lower-limb-only), and sensor configuration density, resulting in substantial heterogeneity. Therefore, the numerical comparisons presented in this and previous sections should be interpreted as indicative of the relative performance positioning of the proposed framework, rather than as evidence of absolute superiority in the absence of fully aligned, controlled benchmarking conditions.

### 4.2. Robustness Against Complex Environmental Interference via Hardware–Software Co-Design

In addition to architectural advantages, the superior performance of the proposed system is largely attributed to the co-design of hardware and algorithms. Many recent deep learning-based pose reconstruction methods (e.g., TIP and TransPose) rely heavily on synthetic datasets generated from SMPL-based models, such as the AMASS dataset. However, simulated environments fail to fully capture real-world sensor imperfections, including bias drift, random walk noise, and geomagnetic disturbances, leading to a significant simulation-to-reality (sim-to-real) domain gap.

In this work, a real-world distributed wireless sensing system was constructed to collect end-to-end training data. Furthermore, an improved adaptive attitude decoupling algorithm was embedded at the preprocessing node. By dynamically adjusting confidence factors for accelerometer and magnetometer measurements at the sensor level, the proposed algorithm is designed to mitigate motion-induced acceleration interference and magnetic field distortions. Ablation results (Table 4 and Table 5) further confirm that, with such high-quality temporal features, the pure Transformer can accurately capture nonlinear kinematic mappings even without a front-end 1D-CNN module. This strategy—performing noise suppression at the hardware level followed by lightweight global feature modeling—provides a novel paradigm for robust wearable sensing system design.

### 4.3. Edge Deployment and Real-Time Performance

To enable truly unconstrained and continuous monitoring beyond laboratory environments, real-time computation capability is essential. In this study, the STM32Cube.AI toolchain was utilized to quantize the floating-point Transformer model and seamlessly deploy it onto a microcontroller platform.

Thanks to the purely Transformer-based architecture dominated by matrix multiplications, which is highly compatible with the dense parallel matrix computation architecture of the built-in NPU (Neural Processing Unit) in the STM32N647 microcontroller, the inference time for a 90-frame sliding window after quantization is reduced to approximately 17 ms. This millisecond-level latency eliminates the need for offline host computation and demonstrates the feasibility of lightweight on-device inference for real-time, low-power wearable applications.

### 4.4. Limitations and Future Work

Despite the promising performance, several limitations remain. First, under highly dynamic activities (e.g., running or jumping), slight sensor displacement caused by soft tissue movement is unavoidable. This introduces low-frequency bias due to relative position changes, which is not yet explicitly compensated by the current model. Future work may incorporate biomechanical constraint-based loss functions to further regularize physically plausible motion reconstruction. To address the prominent roll-angle (Roll) errors observed during high-load movements such as squats, future work will adopt a two-fold improvement strategy. On the hardware side, ergonomically optimized anti-slip cross straps or custom lightweight rigid fixtures will be introduced to achieve more secure fixation of the IMU at anatomical locations less affected by soft tissue deformation (e.g., above the lateral malleolus bony landmark), thereby mitigating soft tissue artefacts (STA). On the algorithmic side, biomechanical constraints will be incorporated into the loss function of the temporal network, such as bilateral symmetry regularization, to leverage physical priors in constraining abnormal divergence in non-stationary axes and further enforce physically plausible motion reconstruction.

Second, the current model is trained and evaluated exclusively on data from healthy subjects without lower-limb impairments. Its generalization capability to pathological gait patterns, such as those associated with knee osteoarthritis (OA) or post-stroke hemiplegia, remains to be validated. Future research will focus on expanding pathological gait datasets and exploring transfer learning strategies for adaptive calibration in patients with movement disorders.

In addition, the subject cohort exhibits a clear gender imbalance (10 males and 2 females), with insufficient consideration of sex-related anatomical differences in the lower limbs. Due to generally wider pelvic morphology and distinct physiological Q-angle in females, their nonlinear kinematic patterns during large-range movements such as squatting and lunging differ significantly from those of males. The current model is therefore overly biased toward male-dominant motion patterns, which limits its generalization robustness on female subjects.

Future work will expand the subject pool and balance the gender distribution. In addition, anatomical geometric priors (e.g., subject-specific Q-angle characteristics) will be incorporated at the algorithmic level as auxiliary inputs, with the aim of mitigating demographic bias and improving motion capture accuracy from a fundamental modeling perspective.

## 5. Conclusions

This paper proposes, designs, and implements a minimal wearable lower-limb kinematic reconstruction system based on distributed edge computing and deep learning. The main contributions of this work are summarized as follows:

1. Hardware architecture: A star-topology distributed motion capture platform was developed, consisting of STM32F103-based preprocessing nodes and an STM32N647-based inference master unit. This design effectively alleviates the bandwidth bottleneck caused by high-frequency data transmission from multiple sensors.
2. Low-level algorithm: An improved adaptive attitude decoupling algorithm incorporating a dynamic confidence factor was derived and embedded at the sensor level; this helps improve the performance of IMU-based attitude estimation under dynamic human motion conditions, providing a more stable temporal feature stream for the downstream reconstruction network.
3. Deep learning model: A Transformer-based architecture was proposed and validated to fuse multimodal temporal inputs (quaternion, acceleration, and angular velocity increments), enabling accurate end-to-end reconstruction of missing lower-limb segment kinematics.
4. Engineering deployment: The floating-point neural network was successfully quantized and deployed onto a resource-constrained microcontroller, achieving fully standalone real-time system operation.

## Figures and Tables

**Table 1:** The error statistics for the inference of five movements—walking, forward leg raise, squatting, lunge stretch, and right leg raise—were evaluated on an independent test set comprising two participants.

Joint	Axis of Rotation	Walk	Forward Leg Raise	Squat	Lunge Stretch	Sides Leg Raise
Pelvis	Roll (deg)	2.41 ± 2.21°	3.56 ± 3.07°	3.64 ± 1.78°	2.64 ± 1.67°	2.70 ± 2.03°
	Pitch (deg)	1.09 ± 1.02°	4.04 ± 3.32°	2.17 ± 1.39°	1.75 ± 1.57°	1.48 ± 1.14°
	Yaw (deg)	1.54 ± 1.28°	2.66 ± 1.92°	1.16 ± 0.81°	2.24 ± 2.20°	4.35 ± 3.98°
Left Thigh	Roll (deg)	3.67 ± 3.04°	2.90 ± 2.76°	3.38 ± 2.10°	2.90 ± 2.97°	3.39 ± 2.78°
	Pitch (deg)	3.16 ± 2.17°	2.59 ± 1.99°	6.09 ± 4.79°	2.11 ± 1.81°	2.76 ± 2.30°
	Yaw (deg)	2.25 ± 2.15°	3.54 ± 3.18°	3.46 ± 2.41°	3.44 ± 3.41°	3.60 ± 2.17°
Left Shank	Roll (deg)	3.08 ± 2.17°	2.51 ± 3.44°	4.45 ± 4.30°	4.04 ± 3.31°	3.12 ± 2.15°
	Pitch (deg)	1.47 ± 1.05°	2.39 ± 2.17°	5.25 ± 4.40°	2.38 ± 1.63°	2.26 ± 1.63°
	Yaw (deg)	3.40 ± 3.13°	2.99 ± 2.76°	6.20 ± 5.02°	4.10 ± 3.74°	3.12 ± 2.95°
Right Thigh	Roll (deg)	1.27 ± 1.03°	2.63 ± 2.25°	3.92 ± 2.86°	3.74 ± 3.25°	2.24 ± 2.31°
	Pitch (deg)	2.11 ± 1.69°	2.16 ± 1.77°	4.44 ± 4.31°	3.05 ± 2.40°	2.94 ± 2.17°
	Yaw (deg)	1.77 ± 1.24°	3.17 ± 2.68°	2.44 ± 1.97°	3.89 ± 2.93°	3.87 ± 3.15°
Right Shank	Roll (deg)	3.84 ± 2.92°	2.67 ± 3.38°	9.94 ± 7.02°	4.17 ± 2.72°	1.54 ± 1.22°
	Pitch (deg)	1.52 ± 1.34°	3.78 ± 1.96°	4.30 ± 3.50°	2.51 ± 1.64°	2.64 ± 1.81°
	Yaw (deg)	2.90 ± 2.62°	3.58 ± 2.39°	5.98 ± 5.01°	4.69 ± 3.11°	1.29 ± 1.07°

**Table 2:** Statistical results of Pearson correlation coefficient (PCC) for lower-limb motion reconstruction across five representative actions (walking, forward leg raise, squat, lunge stretch, and right leg lift) on an independent test set.

Joint	Axis of Rotation	Walk	Forward Leg Raise	Squat	Forward Lunge Stretch	Sides Leg Raise
Pelvis	Roll (deg)	0.759	0.871	0.592	0.943	0.855
	Pitch (deg)	0.433	0.664	0.584	0.867	0.460
	Yaw (deg)	0.617	0.352	0.048	0.842	0.856
Left Thigh	Roll (deg)	0.753	0.810	0.968	0.941	0.784
	Pitch (deg)	0.032	0.401	0.530	0.586	0.116
	Yaw (deg)	0.905	0.888	0.977	0.975	0.241
Left Shank	Roll (deg)	0.911	0.423	0.948	0.882	0.011
	Pitch (deg)	0.768	0.891	0.835	0.835	0.726
	Yaw (deg)	0.929	0.561	0.948	0.888	0.209
Right Thigh	Roll (deg)	0.936	0.847	0.977	0.894	0.829
	Pitch (deg)	0.688	0.818	0.848	0.922	0.153
	Yaw (deg)	0.937	0.908	0.970	0.961	0.344
Right Shank	Roll (deg)	0.856	0.391	0.965	0.833	0.363
	Pitch (deg)	0.888	0.821	0.790	0.916	0.628
	Yaw (deg)	0.896	0.311	0.972	0.776	0.242

**Table 3:** Performance comparison between the proposed method and existing representative approaches based on the number of IMUs, placement, and model architecture. The accuracy is represented by the mean absolute error (MAE) in degrees. Note that all errors are strictly evaluated on the level walking task to maintain objective comparability.

Studies	Number of IMUs	IMU Placement Positions	Model	Accuracy (deg)
Tong Li et al. [31]	2	L/R shank	7-segment model	5.70°
Yifeng Jiang et al. [32]	6	pelvis, head, L/R arm/shank	Transformer	12.19°
Vincent Hernandez et al. [23]	5	pelvis, L/R thigh/shank	DeepConvLSTM	3.60°
Luke Sy et al. [11]	3	pelvis, L/R shank	Constrained Kalman Filter	16.10 ± 3.20°
Ours	3	pelvis, L/R shank	Transformer	2.41°

**Table 4:** Ablation study results of the minimal 3-IMU network under the level-ground walking (Walk) task, reporting mean absolute error (MAE ± standard deviation), 95% confidence intervals, and statistical significance tests across the three anatomical axes of each lower-limb segment.

Movement		Walk				
Joint		Pelvis	Left Thigh	Left Shank	Right Thigh	Right Shank
CNN– Transformer	Roll	2.23 ± 1.96° *** [2.15°, 2.31°]	3.06 ± 2.57° *** [2.96°, 3.16°]	2.25 ± 2.19° *** [2.17°, 2.33°]	1.61 ± 1.75° *** [1.54°, 1.68°]	4.41 ± 3.36° *** [4.28°, 4.54°]
	Pitch	1.11 ± 1.06° *** [1.07°, 1.15°]	2.58 ± 1.89° *** [2.51°, 2.65°]	1.69 ± 1.34° *** [1.64°, 1.74°]	2.21 ± 1.84° *** [2.14°, 2.28°]	1.66 ± 1.92° *** [1.59°, 1.73°]
	Yaw	1.79 ± 0.97° *** [1.75°, 1.83°]	1.79 ± 1.09° *** [1.75°, 1.83°]	2.61 ± 1.69° *** [2.55°, 2.67°]	2.07 ± 2.50° *** [1.98°, 2.16°]	3.29 ± 2.39° ** [3.20°, 3.38°]
Pure- Transformer	Roll	2.41 ± 2.21° [2.33°, 2.49°]	3.67 ± 3.04° [3.56°, 3.78°]	3.08 ± 2.17° [3.0°, 3.16°]	1.27 ± 1.03° [1.23°, 1.31°]	3.84 ± 2.92° [3.73°, 3.95°]
	Pitch	1.09 ± 1.02° [1.05°, 1.13°]	3.16 ± 2.17° [3.08°, 3.24°]	1.47 ± 1.05° [1.43°, 1.51°]	2.11 ± 1.69° [2.05°, 2.17°]	1.52 ± 1.34° [1.47°, 1.57°]
	Yaw	1.54 ± 1.28° [1.49°, 1.59°]	2.25 ± 2.15° [2.17°, 2.33°]	3.40 ± 3.13° [3.28°, 3.52°]	1.77 ± 1.24° [1.72°, 1.82°]	2.90 ± 2.62° [2.80°, 3.00°]
CNN–LSTM	Roll	2.30 ± 1.98° *** [2.23°, 2.37°]	3.06 ± 2.41° *** [2.97°, 3.15°]	3.01 ± 2.94° *** [2.90°, 3.12°]	1.65 ± 1.56° ** [1.59°, 1.71°]	4.45 ± 5.61° ns [4.24°, 4.66°]
	Pitch	1.28 ± 1.18° *** [1.24°, 1.32°]	2.79 ± 2.55° *** [2.69°, 2.89°]	1.65 ± 1.56° ns [1.59°, 1.71°]	2.46 ± 1.81° *** [2.39°, 2.53°]	1.94 ± 2.33° ns [1.85°, 2.03°]
	Yaw	1.77 ± 1.37° *** [1.72°, 1.82°]	2.04 ± 1.73° *** [1.98°, 2.10°]	4.16 ± 3.67° ns [4.02°, 4.30°]	2.85 ± 2.75° *** [2.75°, 2.95°]	3.53 ± 3.51° * [3.4°, 3.66°]
Pure-LSTM	Roll	3.07 ± 2.31° *** [2.98°, 3.16°]	2.75 ± 2.69° *** [2.65°, 2.85°]	3.97 ± 2.84° *** [3.86°, 4.08°]	3.94 ± 2.86° *** [3.83°, 4.05°]	7.42 ± 7.60° *** [7.13°, 7.71°]
	Pitch	1.48 ± 1.28° *** [1.43°, 1.53°]	2.46 ± 1.57° *** [2.4°, 2.52°]	2.15 ± 2.04° *** [2.07°, 2.23°]	2.25 ± 1.71° *** [2.19°, 2.31°]	1.84 ± 1.97° *** [1.77°, 1.91°]
	Yaw	2.01 ± 1.56° *** [1.95°, 2.07°]	3.18 ± 2.76° ns [3.08°, 3.28°]	5.18 ± 4.50° *** [5.01°, 5.35°]	3.75 ± 3.66° *** [3.61°, 3.89°]	7.87 ± 6.14° *** [7.64°, 8.10°]

Note: The results are reported as mean absolute error ± standard deviation (MAE ± SD), with 95% confidence intervals of the MAE shown in square brackets. Statistical significance marks (*) are determined using the proposed Pure Transformer model (Ours) as the reference baseline, against three other baseline methods via frame-wise paired-sample t-tests. Here, *** indicates a highly significant difference (*p* < 0.001), ** indicates *p* < 0.01, * indicates *p* < 0.05, and ns (non-significant) denotes the absence of a statistically significant difference (*p* ≥ 0.05).

**Table 5:** Ablation study results of the minimal 3-IMU network under the squat (Squat) task, reporting mean absolute error (MAE ± standard deviation), 95% confidence intervals, and statistical significance tests across the three anatomical axes of each lower-limb segment.

Movement		Squat				
Joint		Pelvis	Left Thigh	Left Shank	Right Thigh	Right Shank
CNN– Transformer	Roll	4.12 ± 2.46° *** [4.03°, 4.21°]	3.08 ± 2.27° *** [2.99°, 3.17°]	5.42 ± 4.51° *** [5.25°, 5.59°]	3.00 ± 2.16° *** [2.92°, 3.08°]	11.00 ± 9.61° *** [10.64°, 11.36°]
	Pitch	2.03 ± 1.37° *** [1.98°, 2.08°]	6.27 ± 4.86° *** [6.09°, 6.45°]	6.11 ± 5.55° *** [5.90°, 6.32°]	6.61 ± 5.25° *** [6.41°, 6.81°]	5.48 ± 4.68° *** [5.3°, 5.66°]
	Yaw	1.42 ± 1.11° *** [1.38°, 1.46°]	3.96 ± 3.24° *** [3.84°, 4.08°]	6.15 ± 5.07° ns [5.96°, 6.34°]	2.81 ± 2.01° *** [2.73°, 2.89°]	5.39 ± 4.97° *** [5.2°, 5.58°]
Pure- Transformer	Roll	3.64 ± 1.78° [3.57°, 3.71°]	3.38 ± 2.10° [3.3°, 3.46°]	4.45 ± 4.30° [4.29°, 4.61°]	3.92 ± 2.86° [3.81°, 4.03°]	9.94 ± 7.02° [9.68°, 10.2°]
	Pitch	2.17 ± 1.39° [2.12°, 2.22°]	6.09 ± 4.79° [5.91°, 6.27°]	5.25 ± 4.40° [5.08°, 5.42°]	4.44 ± 4.31° [4.28°, 4.6°]	4.30 ± 3.50° [4.17°, 4.43°]
	Yaw	1.16 ± 0.81° [1.13°, 1.19°]	3.46 ± 2.41° [3.37°, 3.55°]	6.20 ± 5.02° [6.01°, 6.39°]	2.44 ± 1.97° [2.37°, 2.51°]	5.98 ± 5.01° [5.79°, 6.17°]
CNN–LSTM	Roll	4.22 ± 2.28° *** [4.13°, 4.31°]	3.02 ± 2.84° *** [2.91°, 3.13°]	3.31 ± 3.08° *** [3.19°, 3.43°]	4.73 ± 2.97° *** [4.62°, 4.84°]	9.75 ± 9.34° ** [9.4°, 10.1°]
	Pitch	3.76 ± 2.54° *** [3.66°, 3.86°]	6.35 ± 4.28° * [6.19°, 6.51°]	4.66 ± 3.89° *** [4.51°, 4.81°]	6.12 ± 4.68° *** [5.94°, 6.30°]	4.58 ± 3.24° *** [4.46°, 4.7°]
	Yaw	1.03 ± 0.81° *** [1.00°, 1.06°]	5.93 ± 5.03° *** [5.74°, 6.12°]	3.46 ± 3.33° *** [3.33°, 3.59°]	3.02 ± 2.75° *** [2.92°, 3.12°]	8.53 ± 5.63° *** [8.32°, 8.74°]
Pure-LSTM	Roll	2.05 ± 1.41° *** [2.00°, 2.10°]	3.61 ± 3.00° *** [3.5°, 3.72°]	4.69 ± 3.90° ns [4.54°, 4.84°]	5.42 ± 2.81° *** [5.31°, 5.53°]	11.94 ± 9.09° *** [11.6°, 12.28°]
	Pitch	3.22 ± 2.30° *** [3.13°, 3.31°]	6.07 ± 4.09° *** [5.92°, 6.22°]	9.89 ± 9.29° *** [9.54°, 10.24°]	7.92 ± 5.09° *** [7.73°, 8.11°]	7.78 ± 6.80° *** [7.52°, 8.04°]
	Yaw	1.99 ± 1.49° *** [1.93°, 2.05°]	6.56 ± 3.90° *** [6.41°, 6.71°]	6.23 ± 5.04° * [6.04°, 6.42°]	11.63 ± 4.85° *** [11.45°, 11.81°]	4.92 ± 3.13° *** [4.8°, 5.04°]

Note: The results are reported as mean absolute error ± standard deviation (MAE ± SD), with 95% confidence intervals of the MAE shown in square brackets. Statistical significance markers (*) are computed using the proposed Pure Transformer model (Ours) as the reference baseline, and are obtained via frame-wise paired-sample t-tests against three baseline methods. Here, *** denotes a highly significant difference (*p* < 0.001), ** denotes *p* < 0.01, * denotes *p* < 0.05, and ns (non-significant) indicates no statistically significant difference (*p* ≥ 0.05).

## Data Availability

The data presented in this study are available on request from the corresponding author due to privacy and ethical restrictions concerning the participants.

## Abbreviations

The following abbreviations are used in this manuscript:

IMU	Inertial measurement unit
NPU	Neural Processing Unit
MAE	Mean absolute error
PCC	Pearson Correlation Coefficient
